# Return Migration of Foreign Students

**DOI:** 10.1007/s10680-015-9360-2

**Published:** 2016-01-25

**Authors:** Govert E. Bijwaard, Qi Wang

**Affiliations:** 1 Netherlands Interdisciplinary Demographic Institute (NIDI-KNAW/University of Groningen), PO Box 11650, 2502 AR The Hague, The Netherlands; 2 Towers Watson, Wuhan, China

**Keywords:** Student migration, Labour dynamics, Marriage formation, Return migration

## Abstract

Using administrative panel data, this paper presents a comprehensive empirical analysis of the return of recent foreign students in the Netherlands. We focus on how individual labour market changes and marriage formation influence their decision to leave. Our model allows for correlated unobserved heterogeneity across the migration, the labour market and the marriage formation processes. The large size of the data permits us to stratify the analysis by five groups based on the country of birth. The empirical analyses reveal that when students become unemployed they leave faster. The effect of finding a job on return is more ambiguous. For students from developed (including EU) countries it hardly affects their return, while students from less developed countries and Antilles/Surinam are more prone to leave after finding a job. Marriage in the Netherlands makes the students more prone to stay.

## Introduction

Among the growing migration population the number of foreign students has increased most rapidly. With the increasing internationalisation of educational programs, students start seeking more and more educational opportunities outside their country of origin. The number of foreign tertiary students in OECD countries has grown 7 % annually from 2000 to 2011 and has reached 4.3 million in 2011. In the Netherlands the number of foreign students has more than doubled from 2000 to 2011. Currently about 9 % of all students at Dutch higher education have a foreign nationality. The inflow of foreign student constitutes about 16 % of the recent total inflow of foreigners into the Netherlands (Bijwaard 2010).

Despite the growing importance of international student mobility there has been relatively little research on student migration. Very often student migration is just regarded as an integral part of migration or as migration of the skilled. Students are, however, a typical group of migrants. They are (very) young, mostly single and much more mobile than labour and family migrants. After graduation many return back to their country of origin. Recent data suggest that only 15–30 % of the foreign students actually decide to stay in their host country (OECD 2013). In the Netherlands about 20 % of the foreign students remain in the country, while 25 % of the labour migrants and 70 % of the family migrants stay (Bijwaard 2010).

Despite the knowledge that many migrations are temporary the majority of the literature on migration still (implicitly) assumes migrations are permanent. The literature has provided numerous explanations for return migration (Borjas and Bratsberg 1996; Dustmann and Weiss 2007). In a recent paper Dustmann and Glitz (2011) formulate a model in which temporary and permanent migrants differ in their strategy of how and where they pursue human capital investments over the life cycle. They argue that migration and education decisions are intertwined. Thus, they are directly linking study and migration behaviour. High incentives to migrate are more efficient investments in education and higher returns to education in the host country. When the additional acquired education in the host country is transferable and study opportunities are better there, a temporary stay abroad may be optimal.

When many migrations are temporary, it is important to know what determines the decision to return. Return migration is intrinsically related to the labour market behaviour of the students and their family formation in the host country. Labour market behaviour is important because the majority of the changes in the socio-economic status of students in the Netherlands are work related and many students find a job soon after graduation. Family formation plays an important role because most students are in their (early) twenties, searching for a spouse, and they are likely to start a family, or at least find a partner, while studying abroad. The influence of labour market experience(s) and marriage formation on subsequent migration behaviour is ignored in the literature on student migration.

The main reason for the limited research on student migration has been the lack of student-specific individual migration data. Usually, the data available are small samples, possibly subject to selectivity and attrition, extracted from surveys. These issues are particularly relevant in studies of migration behaviour since survey attrition usually confounds return migration. The data for this paper are from a unique administrative panel for the entire population of recent immigrants to the Netherlands covering the years 1999–2007. This Dutch immigrant register is based on the legal requirement for immigrants to register with the authorities upon arrival. A feature of our data is the administrative report of the immigration motive (consistent with the visa status at entry). This enables us to focus explicitly and exclusively on 42,730 foreign students. The data contain information on the (day-exact) timing of migration moves to and from the Netherlands, the timing of labour market (and student status) changes and on the timing of marriage formation (while the migrant is registered in the Netherlands). Several other official registers are linked to this immigrant register by Statistics Netherlands, such as the social benefit and the income register (used by the tax authorities).

We address the impact of labour market experience and family formation on migration duration in a novel way that takes the individual timing of labour market changes and (possible) family formation into account, and that controls for the correlation between the potential endogenous labour market and marital status of the migrant and the return decision. To this end we extend the model of Bijwaard et al. (2014), who consider the impact of the individual labour market processes on return, to include (1) a third (studying) status and (2) the family formation process. These processes are interdependent both through observed and unobserved factors. In particular, we estimate the effects of the timing of (un)employment and marriage formation on the hazard of return migration. Given the substantial heterogeneity among foreign students from different origins and the corresponding variation in the immigration policies that influence the movement of students, we separate the students by country of origin groups: (1) EU 15 (including EFTA); (2) new EU, joined the EU in 2004 or 2006; (3) developed countries (DC); (4) less developed countries (LDC); and (5) the former Dutch colonies Antilles and Surinam.

The outline of the paper is as follows. Section 2 reviews previous literature on student and return migration. In Sect. 3 we present some background information regarding studying and entry into the Netherlands. The administrative data we are using for our empirical analyses are described in Sect. 4. The econometric model is set out in detail in Sect. 5. In particular, we specify the labour market, marriage formation and migration processes, and elucidate the role of unobservable heterogeneity. The empirical results are presented in Sect. 6. Finally, Sect. 7 concludes.

## Research on Student Migration and Return

Student migration is connected to human capital theory and regarded as an investment in human capital (Mixon and Hsing 1994). Dustmann and Glitz (2011) formulate an optimal life cycle location model in which migration decisions and decisions about human capital investments are intertwined [see for a similar model Dustmann et al. (2011)]. Migrations are explained as decisions that respond to where human capital can be acquired most efficiently and where the return to human capital is the highest. If human capital accumulation is relatively easier in the host country, this can motivate a temporary stay abroad. Human capital accumulation can take place both through formal education and work experience. As argued by Co et al. (2000) this accumulation will allow the person to enter the home country wage distribution at a relatively higher point upon return, which, even though the home country could have a lower average wage level, will leave the person better off.

Following this argument, spending time abroad studying, can be a way of gaining a competitive edge. This would induce students only to stay for a short period in the host. Dustmann and Glitz (2011) even argue that acquisition of education may be the sole reason for a migration. Implying that the students return after graduation. Empirical evidence on the returns of foreign education shows a wage gain of around 5 % for students from Norway (Wiers-Jenssen and Try 2005) or from Netherlands (Oosterbeek and Webbink 2011) and around 20 % for students from Mexico (Palifka 2003). This seems to imply that returning to the home country is more profitable for students from less developed countries, than from developed countries.

On the other hand, completion of education in the host country enhances the migrant’s host country-specific human capital, thereby facilitating the participation in the host country’s labour market. This may lead to less return. Working after graduation in the host will also improve human capital, which enhances the opportunities in the home country and leading to faster return. Furthermore, it is, due to visa restrictions, for some students more easy to stay than for others. Students from developed countries, especially from EU countries, are less restricted and are therefore more likely to stay after finding employment. However, it is not clear from the start whether foreign students are just coming to enhance their education to obtain higher wages in their home country or that they have the intention to stay and continue working after graduation.

In line with these arguments Rosenzweig (2006) has formulated two competing models for student migration. According to the *school-constraint* model foreign students come from countries with high returns to education but with few domestic opportunities to invest in human capital. Then, students seek training in other countries with the ultimate goal of returning to their home country and reaping the rewards of the high return to education. According to the *migration* model students will acquire schooling abroad as means of entering and staying in the host country when the return to education are low in their home country. In this case, students are simply escaping the low wages at home in search for higher income. In line with the latter case, students choose to study abroad to gain access to the labour market opportunities in the host country. If most students follow the migration model, we expect that the students are more prone to stay after finding a job (and more prone to leave when they become unemployed). If the school-constraint model is dominant, employment would hardly affect return. Dustmann and Glitz (2011) argue that the school-constraint model applies to most students, in the way that most students just study abroad to enhance their education (while their domestic opportunities are not necessarily constraint). We expect that the school-constraint model is more likely to apply for students from LDC countries and Antilles/Surinam, as the opportunities to get higher education are limited there. We would find that these students return fast after graduation. Still they may stay a little bit longer to enhance their human capital even further. Students from developed countries (including the EU) are obviously not school-constraint. Still it is very likely that they will increase their possibilities back home by studying abroad.

Bijwaard (2010) finds high return rates of foreign students coming to the Netherland. The return rate of foreign students is a key issue analysed by Rosenzweig (2008). He finds that the mobility of students can be explained by the same factors that explain international migration in general, higher wages. Bratsberg (1995) provides evidence that the return rate of foreign students from the USA depends on the education level in their home country. When the educational attainment of a student exceeds the average education level in the home country or when the return to education in the home country is higher, a student is more likely to return to the home country. Gibson and McKenzie (2011) find, on the basis of the analysis of migration decisions of top performers form three Pacific countries, that the decision to return is strongly linked to family and lifestyle reasons rather than to the income opportunities in the different destination countries. This implies that marriage during study in the Netherlands would have a large impact on further migration decisions. Marrying a native would make the student more likely to stay in the Netherlands, but the couple might also move to the country of origin of the foreign student. Marrying another foreign student is more likely to induce out-migration to either country of origin. We acknowledge the importance of family on return by allowing that, next to labour market changes, marriage formation also influences the return decision of students.

Enrolment in education affects the timing and occurrence of marriage formation. On the one hand, longer enrolment decreases women’s gain from marriage (Becker 1991) and delays the transition to economic stability typically needed for establishing a union (Oppenheimer 1988; Liefbroer and Corijn 1999), while on the other hand individuals who continue to enhance their human capital have better economic prospects and are therefore more attractive on the marriage market. According to Becker (1991) it could be argued that people marry to maximise their expected well-being. They decide to marry when it brings them higher utility than remaining single. This helps to explain why partners tend to come from similar socio-economic backgrounds. Consequently, students are likely to marry with fellow students, who have similar age, intelligence and education. Thus, international students are very likely to find their spouse while staying in the host country. Union formation also affects schooling behaviour and labour market dynamics. In a marriage individual choices regarding ending school or changing jobs are also based on the implications of these decisions on the well-being of the spouse. In fact, both the marriage formation process and the schooling/labour market process are most likely mutually dependent (Boulier and Rosenzweig 1984; Coppola 2004), in the sense that both observed and unobserved characteristics simultaneously determine individual choice concerning union formation, educational attainment and labour market status.

One important aspect affecting immigrant behaviour is that economic decisions related to the labour market are usually made in conjunction with return migration decisions. Thus, the return decision might be endogenously determined with adverse or positive labour market events such as the occurrence of (un)employment spells. In the literature, we find evidence for the influence of individual labour market changes on the return decision. Kırdar (2009) shows that the effect of unemployment spells on return depends on unemployment durations. Bijwaard et al. (2014) find, using a model that accounts for the endogeneity of labour market changes, that for labour migrants unemployment induces return and re-employment makes the migrants more prone to stay. We extend their model to include marriage formation and transitions from studying to labour market participation which are mutually interdependent with all the other processes.

## Institutional Setting

The Netherlands provides international students the opportunity to study at an institution of higher education. The student needs to comply with the admission requirements as set out in the Dutch Higher Education and Research Act (Wet op het hoger onderwijs en wetenschappelijk onderzoek, WHW). Furthermore to be granted entry into the Netherlands, the student must subsequently comply with the conditions as specified in the Aliens Act. Note that, in the Dutch education system, doctoral candidates are not considered students but researchers, i.e. employees. In our data, we only observe the main source of income (in the Netherlands) in five categories: employee, self-employed, receiving social benefits, receiving student grants and no income (from Dutch sources). It is therefore not possible to distinguish PhD students from other employees.^1^

When an international student wishes to enrol at a Dutch educational institution, admission will be assessed on the basis of the students previous qualifications, the content of the programme completed, the (recognised) diplomas obtained and the language skills geared to the study programme for which admission is requested. Proficiency in English is a compulsory requirement which is tested by means of a language test. International students who have obtained their qualifications in a country where English is the language of tuition and the official working language are exempted from this test. The student must show proof of (provisional) admission to an educational institution funded or designated by the Ministry of Education. The institution must have signed the Code of Conduct and have been entered in the register of the Education Implementation Service. The Code guarantees the quality of the institution’s programmes and its student recruitment, selection and counselling procedures.

Students from non-EU countries who wish to enter the Netherlands for a longer period must apply for a Regular Provisional Residence Permit (MVV) before travelling to the Netherlands. An MVV is a visa for a stay longer than 90 days. The MVV grants entry into the Netherlands and enables her/him to apply for a residence permit for an intended stay for more than 3 months. The MVV requirement does not apply to nationals of the EU/EEA, Australia, Canada, Japan, New Zealand, USA, South Korea and Switzerland. After entry, the MVV is valid for a maximum of 3 months. The student visa is valid for 1 year (or less if the student applies for a shorter study period) and is renewable.

Students from non-EU countries must first satisfy a number of general conditions. These conditions include that the student does not pose a threat to public order or national security and that he has sufficient means of existence. With regard to international students, sufficient means of existence implies that they can pay for their studies and living expenses in the Netherlands independently. For the academic year of 2011/2012, the standard for a student attending higher education is €795 a month. This amount is exclusive of tuition fee.

International students holding a regular residence permit for the purpose of study are permitted to work up to a maximum of 10 h a week, or in the months of June, July and August not more than 40 h a week, in addition to attending the study programme. The student who wishes to work must, however, first ensure that the employer has applied for a work permit. Immigrants from the EU 15 can move freely on the Dutch labour market, as can, since 2004, immigrants from the new EU except for Bulgarians and Rumanians. All non-EU migrants need a work permit (the MVV or Regular Provisional Residence Permit). LDCs and DCs differ in that immigrants from these DCs are exempted from obtaining this MVV before entry.

To bring in a (foreign) partner (either current or future), the partner has to apply for a temporary residence permit. The minimum age for the partner is 18 (family reunification) or 21 (family formation). The sponsor requesting to bring in the partner can be someone with a Dutch nationality or with a foreign nationality holding a residence permit. The sponsor must have sufficient income. This income must have been available for at least 1 year. The minimum level of income for family reunification is 100 % of the level of social assistance for a couple, and the minimum income level for family formation is the minimum wage (which was raised in 2004 to 120 % of the minimum wage). The same rules apply both when a foreign student is the dependent partner in a union with another foreigner and when the sponsor is somebody with a Dutch nationality.

## Data

All legal immigrations of non-Dutch citizens to the Netherlands are registered in the Central Register Foreigners (Centraal Register Vreemdelingen, CRV), combining information from the Immigration Police (Vreemdelingen Politie) and the Immigration and Naturalization Service (Immigratie en Naturalisatie Dienst, IND). Those immigrants who want to stay longer than two-thirds of the next 6 months must notify local population register, municipality, after their arrival in the Netherlands. The administration also records the migration motive of every migrant. The motive is usually coded according to one’s visa status; otherwise it is reported by the immigrant during registration in the population register. Here we focus on migrants who report to migrate as students. We restrict the data to those reported students who had started studying within 3 months after their arrival. Finally, we excluded the students who were married at arrival, about 1.9 %, to avoid initial selection problems in the effect of marriage on return. We end up with 42,730 students who entered the Netherlands between early 1999 and the end of 2007.

Statistics Netherlands has linked the immigration register to the Municipal Register of Population (GBA) and the Social Statistical Database (SSD). The GBA contains basic demographic information of every immigrant, like birthdate, gender, marital status and country of origin. The SSD records monthly information of the individual’s labour market status, income, industry sector, housing and household situation.

The labour market status is defined by the Social Economic Category (SEC), a classification used by Statistics Netherlands based on the main source of income. For somebody with multiple sources of income, like a student with a part-time job or doing an internship, this classification can be misleading. Note that many (non-EU) students are only allowed, implied by their visa, to do small jobs during their studies. When the earnings of such a small (student) job exceeds the amount of student grant/scholarship the student receives, his/her SEC status will change from student to employee even when the student is still studying.

To correct for such spurious labour market status changes we made some data adjustments on short-term employment spells in between study spells. It is reasonable to assume that these short employment spells are just spare-time jobs while studying. These very short employment spells would confound our estimations implying a very dynamic labour market behaviour. Hence, we remove the employment spells of <3 months in between two study spells by assuming that the student remains studying during such a spell. This reduced the number of employment spells from 17,811 to 14,074 (i.e. a reduction of 21 %).^2^

We group our data based on the country of birth of the foreign student. This is strongly related to the visa restrictions students face when trying to study in the Netherlands (see Sect. 3). We distinguish five country of origin groups (1) EU 15 (including EFTA); (2) new EU, joined the EU in 2004 or 2006; (3) DC; and (4) LDC. Finally, we consider students from the former Dutch colonies (Dutch) Antilles and Surinam separately. These countries still have strong ties with the Netherlands and have only limited local study possibilities. Students from LDCs are the largest group with 39 % of the students.

### Summary Statistics

Table 1 provides some descriptive statistics of the foreign students by country group. A slight majority of the students is female. The average age of the students at entry is 22, with students from developed countries on average older and from Antilles/Surinam on average younger. For most groups we observe an increasing inflow of students over the years. Note that the students from the Antilles and Surinam differ substantially from the other students, with two-thirds of the students younger than 20 and a decreasing inflow over the years.

**Table 1:** Sample distribution by region of origin

	EU 15	New EU	DC	LDC	Antilles and Surinam
Female	57.6 %	59.4 %	53.0 %	49.2 %	54.4 %
Interethnic	2.2 %	0.2 %	4.6 %	1.3 %	7.4 %
Dutch parent	2.4 %	0.1 %	2.7 %	0.7 %	2.4 %
Age at entry					
Aged 18–20	33.2 %	26.6 %	18.2 %	28.5 %	66.5 %
Aged 21–24	47.9 %	54.8 %	40.9 %	40.7 %	27.8 %
Aged 25–29	16.7 %	16.4 %	30.6 %	22.0 %	4.6 %
Aged 30–34	1.7 %	1.9 %	7.5 %	6.2 %	0.6 %
Aged ≥35	0.6 %	0.3 %	2.7 %	2.6 %	0.5 %
Average age	22.2	22.4	24.4	23.3	20.2
Year of entry					
1999	3.3 %	1.5 %	2.0 %	2.3 %	12.4 %
2000	3.4 %	1.7 %	3.0 %	4.2 %	13.1 %
2001	4.5 %	3.2 %	3.8 %	7.4 %	13.5 %
2002	5.5 %	7.5 %	6.5 %	11.0 %	13.2 %
2003	9.1 %	9.9 %	10.6 %	14.4 %	12.2 %
2004	14.1 %	14.8 %	15.8 %	13.4 %	9.1 %
2005	16.7 %	19.7 %	20.4 %	14.4 %	7.4 %
2006	20.5 %	19.8 %	19.9 %	15.8 %	9.3 %
2007	22.9 %	21.9 %	18.0 %	17.2 %	9.8 %
*N*	12,124	3375	1998	16,695	8538

Tables 2 and 3 provide some insight into the migration, study, labour market and relationship dynamics. In Table 2 we consider the incidence of return migration and, conditional on returning, the length of stay and the length of study. Note that the group of ‘stayers’ includes permanent migrants and temporary migrants who have not yet returned. Hence, many foreign students, having arrived predominantly during the last 3 years of our observation window, are expected to exhibit a high proportion of incomplete migration spells. This is borne in the data, since the share of stayers is above 65 % for all students.

**Fig. 1:**
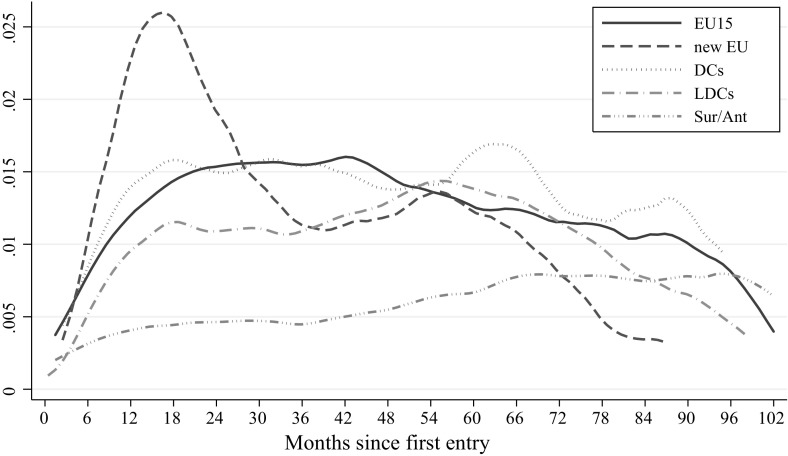
Smoothed Nelson–Aalen return hazard rates

Restricting to the students who have left we observe that students from the EU 15 and less developed countries stay on average for more than 2 years. Students from Antilles/Surinam stay much longer, more than a quart remains for at least 5 years in the Netherlands. The smoothed Nelson–Aalen hazard rates of leaving the Netherlands, presented in Fig. 1, show clear differences among the region of origin of the students.^3^ Students from new EU countries have the highest probability to return and students from Antilles/Surinam the more likely to stay. The hazard functions also show that the timing of return differs substantially among the student groups.

**Table 2:** Descriptive dynamics: migration and study spells

	EU 15	New EU	DC	LDC	Antilles and Surinam
Stayer^a^	69.0 %	65.2 %	66.4 %	69.0 %	74.8 %
Length of stay at return migration					
<6 months	9.4 %	5.4 %	9.3 %	3.1 %	7.8 %
6–12 months	19.1 %	25.9 %	23.9 %	18.7 %	8.4 %
12–18 months	15.4 %	23.1 %	17.0 %	16.2 %	7.5 %
18–24 months	14.7 %	22.0 %	15.5 %	14.3 %	8.3 %
2–3 years	18.5 %	13.3 %	15.0 %	17.8 %	15.0 %
3–4 years	11.7 %	5.0 %	10.2 %	13.5 %	13.3 %
4–5 years	5.8 %	3.6 %	3.5 %	9.7 %	13.4 %
>5 years	5.3 %	1.8 %	5.5 %	6.7 %	26.4 %
Average (months)	24.7	19.5	22.9	28.2	40.8
Study dynamics					
Always studying^b^	66.4 %	54.5 %	53.2 %	50.7 %	54.1 %
At least once employed	16.6 %	18.5 %	12.7 %	20.8 %	38.8 %
At least once no work	24.8 %	35.4 %	41.4 %	40.3 %	23.5 %
Length of study till emigration, work or no work					
<6 months	14.2 %	14.3 %	13.6 %	10.0 %	18.9 %
6–12 months	23.7 %	33.1 %	30.6 %	22.7 %	14.3 %
12–18 months	12.9 %	21.3 %	12.4 %	12.9 %	9.7 %
18–24 months	14.4 %	11.3 %	18.2 %	18.8 %	8.4 %
2–3 years	16.0 %	9.3 %	12.5 %	15.5 %	13.2 %
3–4 years	10.5 %	6.4 %	7.6 %	10.5 %	10.9 %
4–5 years	4.6 %	2.7 %	3.1 %	6.1 %	10.2 %
>5 years	3.7 %	1.7 %	2.1 %	3.6 %	14.4 %
Average (months)	22.0	17.4	19.4	23.4	29.6
Average at departure^c^	22.2	15.5	17.1	21.6	29.6

^a^Stayers are migrants who remain in the country till the end of the observation period

^b^Percentage of migrants that is studying through the whole stay in the country

^c^Average study length at departure when studying till the end

**Fig. 2:**
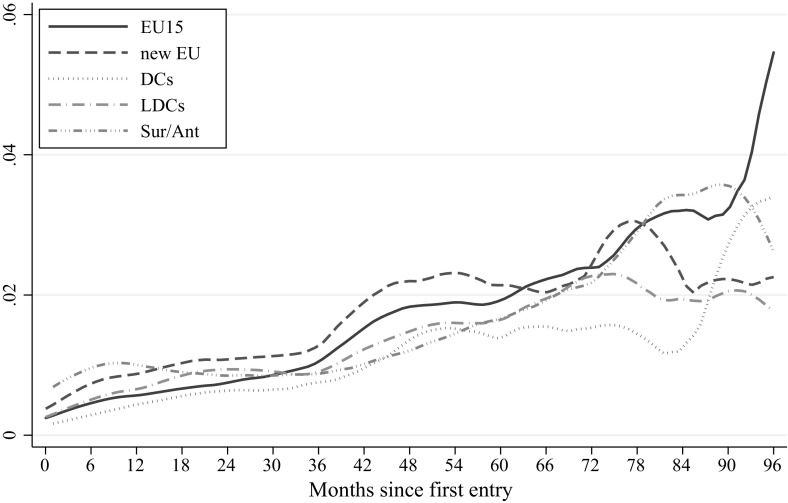
Smoothed Nelson–Aalen hazard rates of entering the labour market

More than half of all students ($\tfrac{2}{3}$ of the EU 15 students) leave the country when their study period ends. However, a substantial minority of the students have labour market experience in the Netherlands (see Table 2). Figure 2 depicts the (smoothed) hazard of entering the labour market. All the groups look very similar. The hazard of finding a job is steadily increasing with the time spend in the Netherlands. Students from the new EU are slightly more likely to start working, and students from developed countries are slightly less likely to start working.

In Table 3 we consider the labour market dynamics in more detail. Since we are mainly interested in the effects of labour market experience and marriage formation on the length of stay in the Netherlands, we focus on the labour market status of the students prior to departure. Only a small fraction of the students leave while they are employed. It is clear from the low percentages of students who leave (un)employed that most students just stay till the end of their study period. It seems that they only immigrate to the Netherlands to acquire education. We test this with our model.

**Fig. 3:**
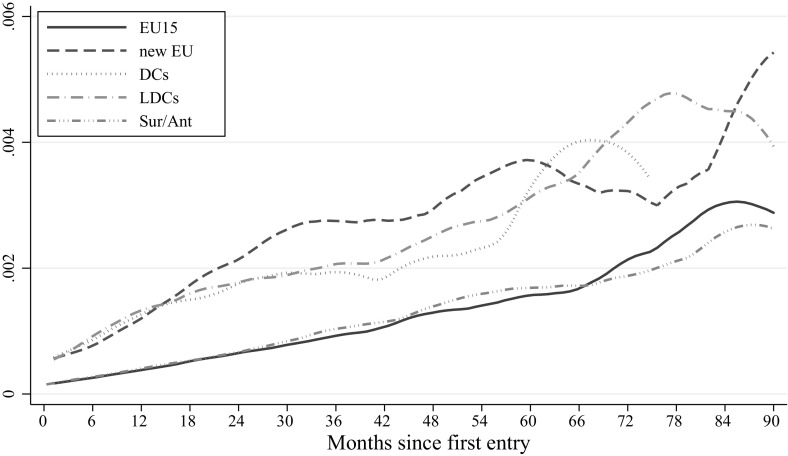
Smoothed Nelson–Aalen hazard rates of getting married (in the Netherlands)

Only a very small fraction of the, original single, students leaves married, 0.2–0.7 %. The smoothed hazard rates in Fig. 3 show that the incidence of getting married is very low, but steadily increasing, for all groups of foreign students. Students from developed countries, both from inside and from outside the EU, are more prone to marry while in the Netherlands. Unfortunately, we do not observe cohabitation but only official marriages. This leads to an underrepresentation of union formation. Cohabitation is rather common in most EU countries. So, we expect that we will have the largest underrepresentation for students from EU 15 countries.

**Table 3:** Descriptive dynamics: labour market and marriage

	EU 15	New EU	DC	LDC	Antilles and Surinam
Employed leaver^a^	1.6 %	1.2 %	1.0 %	1.3 %	3.6 %
Length of observed employment spell(s)^b^					
<1 months	6.9 %	7.0 %	7.0 %	8.0 %	5.6 %
1–3 months	25.3 %	25.3 %	24.3 %	27.2 %	24.3 %
3–6 months	22.0 %	27.8 %	27.0 %	20.8 %	21.0 %
6–12 months	25.7 %	24.1 %	25.9 %	25.5 %	27.9 %
1–2 years	14.2 %	9.2 %	12.4 %	12.8 %	13.3 %
>2 years	5.9 %	6.7 %	3.2 %	5.8 %	8.0 %
Average (months)	8.4	8.2	7.2	8.0	9.4
No work dynamics					
No work leaver^c^	3.7 %	7.0 %	5.4 %	5.6 %	4.9 %
Length of observed no work spell(s)^d^					
<1 months	11.6 %	6.7 %	8.4 %	10.1 %	15.2 %
1–3 months	34.5 %	30.1 %	29.3 %	29.1 %	34.5 %
3–6 months	21.6 %	33.6 %	19.5 %	21.1 %	19.7 %
6–12 months	18.7 %	18.0 %	24.9 %	21.0 %	18.1 %
1–2 years	10.8 %	9.3 %	14.8 %	13.6 %	8.3 %
>2 years	2.8 %	2.3 %	3.0 %	5.2 %	4.2 %
Average (months)	6.1	6.0	7.2	7.5	6.1
Marriage dynamics					
Married leaver^e^	0.2 %	0.3 %	0.7 %	0.4 %	0.6 %
Length of observed single spell^f^					
Average (months)	38.1	28.3	27.5	33.0	49.2
Length of marriage spell till departure					
Average (months)	21.9	16.4	20.3	16.4	21.1

^a^Percentage of students who are employed at the moment of departure

^b^Employment spell(s) that ends in departure, or no work

^c^Percentage of students who are not working (nor studying) at the moment of departure

^d^No work spell(s) that ends in departure, or employment

^e^Percentage of students who are married at the moment of departure

^f^Single spell that ends in marriage

## Methodology

A major methodological concern with the empirical analysis of the impact of labour market experience and marriage formation in the host country on return migration decisions is that all these processes depend on student characteristics. This implies that any observed relationship between individual labour market changes or marriage formation and return migration may be caused by unobserved factors that influence both the labour market dynamics, the marriage formation and the return migration decision. For example, a finding that female students have shorter migration durations in the host country may not imply that gender causes students to leave fast. Rather, it may be induced by other factors of female students which make them to stay for a shorter period. Labour market behaviour in the host country plays here a central role. Unemployment has been shown to affect the return decision (Bijwaard et al. 2014; Kırdar 2009). It is therefore imperative to account for interdependence between labour market changes (and marriage formation) and return. We use a ‘timing-of-events model’ (Abbring and Berg 2003), which explicitly controls for the strong correlation between labour market changes and the decision to return (Bijwaard et al. 2014), to account for this interdependence.

Thus, we seek to identify the effect of labour market and family formation dynamics on foreign students’ decision to leave. Let $T_{\mathrm{m}}$ denote the time (since entry into the Netherlands) the immigrant emigrates from the host country, $T_{\mathrm{s}}$ the time a study spell ends in the host country, $T_{\mathrm{e}}$ the time an employment spell ends, $T_{\mathrm{u}}$ the time an unemployment spell ends, and $T_{\mathrm{mar}}$ the time a migrant marries in the host country (all students are single at entry). A study spell can end in either employment or unemployment (or departure).

The durations of a study spell ending in employment and unemployment spells are denoted by $\delta _{\mathrm{se}}(t)$ and $\delta _{\mathrm{su}}(t)$. Similarly, the durations from employment to unemployment is denoted by $\delta _{\mathrm{eu}}(t)$ and from unemployment to employment by $\delta _{\mathrm{ue}}(t)$. In order to keep track of labour market and marriage events, we also define the associated time-varying indicators: the indicator $I_{\mathrm{u}}{(t)}$ takes value one if the migrant is unemployed at time *t*,^4^ $I_{\mathrm{e}}(t)$ indicates that the immigrant is employed, and $I_{\mathrm{mar}}(t)$ indicates that the immigrant is married.

**Fig. 4:**
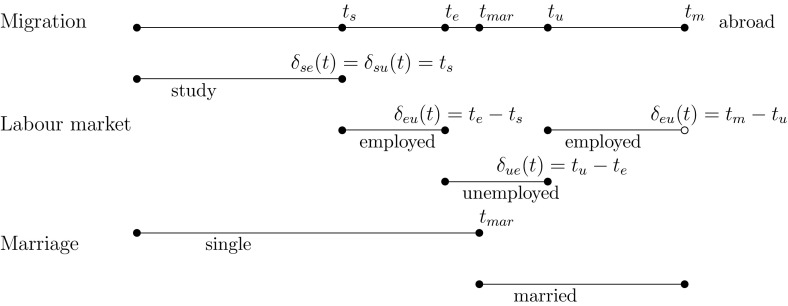
Migration, labour market and marriage dynamics

In Fig. 4 we depict the labour market, marriage and migration dynamics of an arbitrary foreign student. The student is (by definition) studying and single at entry. After a study period of $t_{\mathrm{s}}$ the student finds a job in the Netherlands. This implies that the time till employment is $\delta _{\mathrm{se}}(t)=t_{\mathrm{s}}$, which is equal to the (censored) time till unemployment, $\delta _{\mathrm{su}}(t)=t_{\mathrm{s}}$. The student is fired at $t_{\mathrm{e}}$. Thus the (first) job lasts for $\delta _{\mathrm{eu}}(t)=t_{\mathrm{e}}-t_{\mathrm{s}}$. After a period of unemployment $\delta _{\mathrm{ue}}(t)=t_{\mathrm{u}}-t_{\mathrm{e}}$, the student finds a new job at time $t_{\mathrm{u}}$. In the meantime the student got married at time $t_{\mathrm{mar}}$, which implies that he/she has been single for $t_{\mathrm{mar}}$. At the moment the student leaves the country, at $t_{\mathrm{m}}$, the employment spell (and the marriage spell) in the Netherlands ends. This implies that the second employment spell was censored and of length $\delta _{\mathrm{eu}}(t)=t_{\mathrm{m}}-t_{\mathrm{u}}$. We assume that all these events also change the incidence of the other events and that the incidence depends on (un)observed individual factors that influence all the events simultaneously.

We consider three different processes: (1) the labour market process, including studying; (2) the process of getting married; and (3) the main process of leaving the country. As the migrant is either studying, employed or unemployed, the labour market process has four possible transitions: study to employment (se), study to unemployment (su), employment to unemployment (eu) and unemployment to employment (ue). Note that all the students are, by definition, studying at entry. So, there is no need to model any initial conditions to enter the first state. The conditional hazards for these transitions all follow mixed proportional hazard (MPH) models and are allowed to be correlated through unobservable heterogeneity terms:

1 $$ \theta _{k}\left. \left( \delta _{k}(t)|t_{\mathrm{mar}}, x_{k}(t), v_{k}\right) = v_{k} \lambda _{k}\left( \delta _{k}(t)\right) \exp \left( x_{k}(t)\beta _{x}^{k} +I_{\mathrm{mar}}(t)\gamma _{\mathrm{mar},k}\right) \right., $$

with $k=\{ \hbox {se}, \hbox {su}, \hbox {eu}, \hbox {ue} \}$. The baseline hazard $\lambda _{k}(\cdot )$, which is common to all individuals, reflects the duration dependence of the particular hazard rate. The exogenous (control) covariates that may explain the labour market transition, $x_{k}(t)$, are possibly time-varying and enter the hazard exponentially, $\exp (x_{k}(t)\beta _{x}^{k})$, which accelerates exits. To accommodate unobserved heterogeneity a positive time-invariant individual-specific random term, $v_k$, multiplies the hazard. Two additional terms are included: $I_{\mathrm{mar}}(t)$ indicates that a student is married at *t* and $\gamma _{{\mathrm{mar}},k}$ captures the effect of marriage on these labour market transition hazards.

Most students are in their 20s, and this age period is generally the onset of family formation. Students at campus or starting their career are prone to find their partner. The hazard of marrying is also of the MPH form and we allow for a direct effect of (un)employment on this transition:^5^

2 $$ \theta _{\mathrm{mar}}\left( t| t_{\mathrm{e}}, t_{\mathrm{u}}, x_{\mathrm{mar}}(t), v_{\mathrm{mar}}\right) =v_{\mathrm{mar}} \lambda _{\mathrm{mar}}( t)\exp \left( x_{\mathrm{mar}}(t)\beta _{x}^{\mathrm{mar}} +I_{\mathrm{e}}(t)\gamma _{e,\mathrm{mar}}+I_{\mathrm{u}}(t)\gamma _{u,\mathrm{mar}}\right) ,$$

with $I_{\mathrm{u}}(t)$ and $I_{\mathrm{e}}(t)$ are the indicators of (un)employment of the student and $\gamma _{e,\mathrm{mar}}$ and $\gamma _{u,\mathrm{mar}}$ capture the effect of these labour market changes on the hazard to get married. Again $\lambda _{\mathrm{mar}}(\cdot )$ captures the duration dependence, $x_{\mathrm{mar}}(t)$ are (possibly time-varying) control variables explaining the hazard of marrying and, $v_{\mathrm{mar}}$ is a positive time-invariant individual-specific random term capturing unobserved heterogeneity.

Finally, the return migration hazard also has an MPH form. The migration hazard is a function of (possibly time-varying) control variables $x_{m}(t)$, labour market changes, $I_{\mathrm{u}}(t)$ and $I_{\mathrm{e}}(t)$, and getting married $I_{\mathrm{mar}}(t)$

3 $$ \theta _m\left( t|t_{\mathrm{u}}, t_{\mathrm{e}}, t_{\mathrm{mar}}, x_m(t), v_m\right) = v_m \lambda _{m}(t)\exp \left( x_m(t)\beta _{x}^m + I_{\mathrm{u}}(t)\gamma _u +I_{\mathrm{e}}(t)\gamma _e + I_{\mathrm{mar}}(t) \gamma _{\mathrm{mar}} \right). $$

Again $\lambda _{m}(\cdot )$ captures the duration dependence and $v_{\mathrm{m}}$ is a positive time-invariant individual-specific random term capturing unobserved heterogeneity.

It is well known that, due to dynamic sorting effects, the distribution of the unobserved heterogeneity among those students who become (un)employed or married at a particular time will differ from its population distribution. Consider, for example, the student to employment process. Students with high $v_{\mathrm{se}}$, e.g. high motivation to become employed, will tend to enter employment earlier than individuals with low $v_{\mathrm{se}}$. If $v_{\mathrm{se}}$ and $v_{\mathrm{m}}$, the unobserved heterogeneity of the return migration hazard, are dependent, then the distribution of $v_{\mathrm{m}}$ for employed students at a given time in the country will differ from the distribution of $v_{\mathrm{m}}$ for students still studying. Similarly, if $v_{\mathrm{m}}$ and $v_{\mathrm{mar}}$ are dependent, then the distribution of $v_{\mathrm{m}}$ among married students will differ from its population distribution. Therefore, one cannot infer the effect of (un)employment or marriage on the return migration from a comparison of the realised durations of those who became (un)employed/married at a particular time with the rest of the population, because one would then mix the effect of (un)employment/marriage on the duration with the difference in the distribution of $v_{\mathrm{m}}$ between these migrants. In this case $I_{\mathrm{e}}(t), I_{\mathrm{u}}(t)$ and $I_{\mathrm{mar}}(t)$ will be endogenous. The same holds for the inclusion of the marriage in the labour market processes and for the inclusion of (un)employment in the marriage process, and therefore all the durations $T_{\mathrm{se}},\ldots , T_{\mathrm{mar}}$ and $T_{\mathrm{m}}$ should be modelled jointly to account for dependence of the unobserved heterogeneity terms.

For the sake of parsimoniousness, we assume that each of the unobserved heterogeneity terms remains the same for recurrent durations of the same type, and we adopt a discrete distribution, i.e. *v* has discrete support $(v_1, \ldots , v_K)$, with $v_{\mathrm{r}}=(v_{\mathrm{se,r}},\ldots ,v_{\mathrm{m,r}})$ and $p_{\mathrm{r}}= \Pr (v=v_{\mathrm{r}})$.^6^

The “timing-of-events” method of Abbring and Berg (2003) implies that the full effects of labour market changes and marriage formation on the return migration hazard, $\gamma _{e}$, $\gamma _{u}$ and $\gamma _{\mathrm{mar}}$ in our framework, have a causal interpretation. This requires that all transition rates are modelled parametrically as mixed proportional hazards, as we have. Identification of the causal effect additionally requires that the so-called no-anticipation assumption holds. The (untestable) no-anticipation assumption requires that migrants do not anticipate entering the labour market (or marriage) by migrating before the anticipated event would occur. Although it can be argued that the no-anticipation assumption is valid, we are cautious in using a casual interpretation of the our effects. Still, the timing-of-events method corrects for possible endogeneity of the labour market and marriage formation processes.

Although in principle the exact date of emigration is known, some migrants do not officially inform the authorities that they are about to leave the Netherlands. However, all citizens (immigrants and natives) are required to register with their municipalities (this is a pre-requisite for many social services and for tax-benefit matters). It is thus clear that any migrant who has no entries in the tax-benefit register and does not appear in the register of another municipality must have left the country. Only the exact date of the departure is unknown. Such non-compliers are periodically identified and removed from the registers by the authorities in a step labelled “administrative removal”. These administrative removals are included among the emigrations. In our data the percentage of administrative removals among the emigrants runs from 14 % for students from Antilles and Surinam to 46 % for students from developed countries (EU 15: 33 %; new EU: 37 % and LDC: 40 %).

We assume that when a migrant is “administrative removed” and has “zero income at the last observed time” implies that the migrant has left *before* the date the administrative removal is recorded and *after* the last date of any observed change in the observed characteristics (e.g. labour market status, housing and marital status). Such limited information is equivalent to *interval-censored* data. For interval-censored data the exact timing of an event is unknown, but it is known that the duration ended in some period of time.

## Estimation Results

We reckon that the demographic factors gender, age at entry, inter-ethnicity and having a Dutch parent influence the decision to return. Macro- and business cycle-factors are captured by the national unemployment rate (changing on a quarterly basis), the national unemployment rate at the moment of entry and the national employment rate at different migration durations. We control for cohort effects by including both the year of entry and the unemployment rate at entry. We assume piecewise constant baseline hazards on six intervals, 0–6, 6–12 months, 1–2, 2–3, 3–5 and more than 5 years, with the first 6 months as reference.

### Impact of Control Variables on Return

Before we turn to the discussion of our main results, the impact of labour market and marriage formation processes on the migration hazard, we briefly discuss the impact of included control variables on the return hazard (see Table 4).^7^ We observed large differences among the country groups. In the empirical literature on migration dynamics it is usually found that females are less mobile than men, we find, in line with Bijwaard (2010), that gender hardly influences departure of foreign students. For labour migrants it is commonly found that both the younger and the older leave faster than those in their prime of their working age. Students are almost all below 30, and we find that the migration hazard is higher for students aged 21–29 (compared to the reference groups of students aged 18–20). For students from less developed countries the migration hazard is also higher for older, beyond 30 years of age, students. Students from Antilles/Surinam and developed countries do not exhibit an age effect on return. Not surprisingly, students with a Dutch parent stay more often (only significant for Antilles/Surinam and EU 15 students). We find rather large cohort effects, especially for students from new EU countries, indicating that the most recent cohorts leave (much) faster. In an economic crisis, captured by a high (national) unemployment rate, it is harder for the students to find a job and this induces them to leave. For EU 15 students this only holds after 6 months of stay. In contrast to these business cycle effects we find that students from new EU and less developed countries are more prone to stay when they arrive in a period with high unemployment. Although this seems to indicate a negative self-selection of students from these countries, this selection might be due to push factors if their home countries are simultaneously in a crisis (which is often the case, especially for the new EU countries) and they prefer to continue studying to avoid unemployment in the home country. Of course, we should remember that their first motive to migrate to the Netherlands was to study and not to work. The moment they graduate (a few years later) the labour market situation usually has changed dramatically, either for the good when the country was in a crisis or for the better when the country was in a boom. So, the unemployment at entry may capture other selection effects. The departure of students from developed countries and from Antilles/Surinam is hardly affected by the business cycle in the Netherlands. The estimated baseline hazard implies a strong positive duration dependence; the longer the students are in the country, the higher the hazard to return, especially for students from new EU countries. The return hazard for students from the Antilles/Surinam only starts increasing 3 years after arrival.

**Table 4:** Estimated impact of control variables on the return migration hazard

	EU 15	New EU	DC	LDC	Surinam/Antilles
Female	−0.062 (0.037)	−0.247^+^ (0.100)	−0.145 (0.095)	0.031 (0.041)	0.113^+^ (0.046)
Age 21–24	0.222** (0.045)	1.127** (0.126)	0.145 (0.134)	0.321** (0.055)	0.107^+^ (0.050)
Age 25–29	0.182** (0.058)	0.744** (0.159)	0.067 (0.143)	0.313** (0.063)	0.039 (0.097)
Age 30–34	−0.016 (0.144)	0.313 (0.435)	0.090 (0.203)	0.561** (0.088)	−0.048 (0.293)
Age >35	0.160 (0.245)		0.180 (0.297)	0.622** (0.123)	−0.602 (0.394)
Interethnic	0.200 (0.139)		0.001 (0.241)	−0.484^+^ (0.223)	0.081 (0.117)
NL parent	−0.373** (0.142)		−0.124 (0.333)	−0.062 (0.262)	−0.482^+^ (0.205)
Unemployment (nat)	0.049 (0.085)	0.619** (0.219)	0.278 (0.237)	0.416** (0.120)	0.084 (0.107)
U at entry	−0.094 (0.086)	−0.868** (0.248)	−0.088 (0.232)	−0.479** (0.110)	−0.266 (0.143)
Year 2000	0.184 (0.121)	−0.320 (0.519)	0.162 (0.365)	0.064 (0.161)	0.095 (0.112)
Year 2001	0.397** (0.148)	−0.223 (0.583)	0.089 (0.425)	0.181 (0.190)	0.192 (0.178)
Year 2002	0.595** (0.134)	0.819 (0.530)	0.478 (0.400)	0.596** (0.168)	0.556** (0.141)
Year 2003	1.052** (0.119)	2.721** (0.471)	0.986^+^ (0.387)	1.607** (0.156)	0.874** (0.130)
Year 2004	1.444** (0.145)	3.206** (0.510)	1.564** (0.463)	2.606** (0.197)	1.125** (0.218)
Year 2005	1.788** (0.140)	4.813** (0.501)	2.097** (0.467)	2.945** (0.189)	1.293** (0.207)
Year 2006–2007	1.802** (0.121)	4.482** (0.485)	2.441** (0.438)	3.031** (0.162)	1.073** (0.153)
Unemployment (nat.) (6–12 months)	0.180^+^ (0.087)	−0.374 (0.203)	0.139 (0.220)	−0.190 (0.121)	0.319^+^ (0.132)
Unemployment (nat.) (1–2 years)	0.183^+^ (0.089)	0.511^+^ (0.214)	0.074 (0.222)	-0.024 (0.120)	0.022 (0.122)
Unemployment (nat.) (2–3 years)	0.282** (0.104)	−0.023 (0.250)	0.342 (0.272)	−0.078 (0.130)	0.015 (0.134)
Unemployment (nat.) (3–5 years)	0.285^+^ (0.111)	−0.389 (0.286)	0.205 (0.298)	−0.096 (0.136)	0.095 (0.135)
Unemployment (nat.) (>5 years)	0.434** (0.125)	−0.351 (0.429)	−0.025 (0.327)	0.042 (0.153)	0.205 (0.121)
Duration dependence					
*α*_2_ (6–12 months)	0.911** (0.080)	2.169** (0.186)	1.088** (0.221)	2.142** (0.117)	−0.014 (0.125)
*α*_3_ (1–2 years)	1.162** (0.086)	3.117** (0.206)	1.384** (0.248)	2.469** (0.122)	0.136 (0.110)
*α*_4_ (2–3 years)	1.423** (0.102)	4.266** (0.268)	1.443** (0.313)	2.620** (0.138)	0.189 (0.121)
*α*_5_ (3–5 years)	1.775** (0.108)	4.903** (0.325)	1.980** (0.343)	3.335** (0.146)	0.253^+^ (0.117)
*α*_6_ (>5 years)	1.982** (0.132)	5.117** (0.459)	2.798** (0.406)	3.898** (0.166)	0.705** (0.114)

^+^ *p* < 0.05; ** *p* < 0.01

### Effect of Labour Market Dynamics and Marriage Formation on Return

The full model, given by the correlated MPH hazards (1)–(3), nests the conventional (M)PH models for the return hazard. The PH model ignores unobservable heterogeneity altogether, $\theta _m ^{\mathrm{PH}}(t|t_{\mathrm{u}}, t_{\mathrm{e}}, t_{\mathrm{mar}}, x_m(t)) = \exp ( x_m(t)\beta _{x}^m + I_{\mathrm{u}}(t) \gamma _u +I_{\mathrm{e}}(t)\gamma _e +I_{\mathrm{mar}}(t)\gamma _{\mathrm{mar}}),$ whereas the MPH model, $\theta _m ^{\mathrm{MPH}}(t|t_{\mathrm{u}}, t_{\mathrm{e}}, t_{\mathrm{mar}}, x_m(t),v_m)= v_m \theta _m ^{\mathrm{PH}}(t|t_{\mathrm{u}}, t_{\mathrm{e}}, t_{\mathrm{mar}}, x_m(t))$ ignores the correlation between $\theta _m$ and the labour market and marriage hazards. To illustrate the consequences of ignoring the endogeneity induced by the correlations between the unobservable heterogeneity terms, we report in Table 5 the estimated effects of labour market dynamics and marriage formation for the PH, MPH and the full model.

**Table 5:** The impact of the labour market and marriage dynamics on return migration hazards

	EU 15	new EU	DC	LDC	Surinam/Antilles
Employment effect, *γ*_*e*_					
PH model	−0.392** (0.069)	−0.478** (0.148)	−0.284 (0.216)	−0.258** (0.068)	0.450** (0.063)
MPH model	−0.142 (0.081)	−0.546** (0.155)	0.070 (0.291)	−0.289** (0.070)	0.491** (0.069)
Correlated model	−0.064 (0.100)	−0.016 (0.241)	−0.307 (0.269)	0.262** (0.095)	0.728** (0.076)
Unemployment effect, *γ*_*u*_					
PH model	0.961** (0.039)	1.149** (0.064)	1.252** (0.090)	1.441** (0.032)	2.206** (0.050)
MPH model	0.843** (0.058)	1.430** (0.084)	0.979** (0.144)	1.588** (0.037)	2.232** (0.063)
Correlated model	0.758** (0.064)	1.194** (0.104)	1.214** (0.133)	1.749** (0.076)	2.118** (0.070)
Marriage effect, *γ*_mar_					
PH model	−0.828** (0.195)	−1.054** (0.286)	−0.408 (0.252)	−0.999** (0.102)	−0.355** (0.131)
MPH model	−0.965** (0.258)	−1.285** (0.315)	−0.149 (0.367)	−1.114** (0.108)	−0.326^+^ (0.163)
Correlated model	−0.882** (0.226)	−0.142 (0.512)	−0.314 (0.285)	−1.212** (0.160)	−0.355^+^ (0.143)

^+^ *p* < 0.05; ** *p* < 0.01

Ignoring the correlations between the processes leads to misleading conclusions about the impact of the labour market and marriage processes on the return migration hazard. Based on the results from a simple PH model we would have concluded that for most foreign students, except those from Antilles/Surinam, finding employment in the Netherlands makes them more prone to stay. Accounting for (uncorrelated) unobserved heterogeneity in an MPH model already removes the significance of the impact of employment on the return hazard for students from the EU 15. Substantial changes in the estimated effects of employment are observed when accounting for correlation between the unobserved terms. The results from the correlated model reveal that students from less developed countries are also more prone to leave after becoming employed. From the correlation of the unobserved heterogeneity terms^8^ we can infer that those who end study fast to work stay longer in the country. Ignoring this correlation induced by unobserved factors that both explain the hazard of finding work and the hazard of leaving the country leads to a negative bias in the estimated impact. It seems that students from LDCs and Antilles/Surinam use employment either to enhance their human capital further to obtain a higher wage back home or to save money before returning.

The impact of unemployment on return only changes slightly when accounting for (correlated) unobserved heterogeneity. For all five student groups we confirm that unemployment leads to faster return. Note that unemployed students usually are not eligible for social benefits such as unemployment insurance payments, since these are conditional on sufficiently long employment. Entering unemployment therefore leaves the student with little income which make them more prone to find a job elsewhere. This unemployment effect is particularly strong for students from Antilles/Surinam, which is in line with the constrained domestic schooling model.

For three of the student groups forming a marriage extends the duration of stay. This marriage effect is particularly strong for students from less developed countries. The strong marriage effect found by the (M)PH models for students from new EU countries completely disappears when we account for correlation between the unobserved factors of the migration and the marriage formation hazard. It seems obvious that students become more attached to the Netherlands after marriage. Unfortunately, we do not have spousal information, neither whether the partner is a native nor what the labour market situation of the partner is. When the partner is also a foreigner, the couple may leave together to a third country (of course, this can also happen when the foreign student marries with a Dutch native). From the insignificant impact of marriage on return for new EU and developed countries we tentatively conclude that they marry less with Dutch natives.

### Heterogeneous Effects

Next we allow for heterogeneity in the impact of (un)employment and marriage on the migration hazard. We allow the effect to vary by gender, age and the business cycle of the Dutch economy (see Table 6).

**Table 6:** Heterogeneity in effect of labour market dynamics and marriage on return

	EU 15	new EU	DC	LDC	Surinam/Antilles
Effect of employment					
Female	0.046 (0.142)	0.163 (0.332)	0.074 (0.516)	0.030 (0.140)	−0.266^+^ (0.124)
Age at entry >25	−0.268 (0.158)	−0.238 (0.469)	−0.576 (0.564)	−0.858** (0.162)	−0.585** (0.220)
Unemployment (nat.)	0.059 (0.086)	0.095 (0.216)	0.171 (0.340)	0.004 (0.090)	0.079 (0.075)
Constant	−0.038 (0.144)	−0.162 (0.316)	−0.095 (0.485)	0.463** (0.125)	0.873** (0.105)
Effect of unemployment					
Female	0.061 (0.086)	0.062^+^ (0.174)	0.071 (0.207)	0.030 (0.079)	−0.130 (0.104)
Age at entry >25	−0.322** (0.107)	−0.178 (0.241)	0.170 (0.214)	−0.710** (0.085)	−0.798** (0.237)
Unemployment (nat.)	0.086 (0.055)	0.219 (0.123)	0.064 (0.133)	−0.024 (0.051)	−0.049 (0.068)
Constant	0.763** (0.086)	1.066** (0.161)	1.078** (0.214)	1.966** (0.097)	2.286** (0.099)
Effect of marriage					
Female	−0.176 (0.483)		−1.311 (0.946)	−0.400 (0.246)	−0.417 (0.290)
Constant	−0.758 (0.394)	−0.027 (0.552)	0.665 (0.822)	−0.935** (0.219)	−0.098 (0.222)

^+^ *p* < 0.05; ** *p* < 0.01

The effect of employment on return is depicted in the first panel of the table. The gender of the student does not significantly change the effect of becoming employed, except for students from the former Dutch colonies. In general older students (students who arrive at a higher age) are more focussed on a stable job and stable residence and are therefore more prone to stay when they find a job (only significant for LDC and Antilles/Surinam students). The business cycle does not significantly change the impact of becoming employed on return.

The effect of unemployment on return is depicted in the second panel of Table 6. Gender only influences the impact of unemployment on return for students from the new EU countries. Female students from these countries have a larger impact of unemployment on return. For most student groups a higher age at arrival reduces the impact of unemployment. However, given the large increase in return due to unemployment, these older students are still more likely to leave when they become unemployed. The business cycle does not significantly change the impact of unemployment on return.

We do not find any significant heterogeneity in the impact of marriage on return, depicted in the third panel of Table 6. This is probably because we observe too few students marrying in the Netherlands.

## Conclusions and Discussion

Despite that international student mobility has increased substantially, little research has focussed on migration behaviour of students. An important issue in researching student migration is that the majority of foreign students only stay temporarily. The process of their return migration is intrinsically related to their life course behaviour in the host country, both on the labour market and on the marriage market. These processes are likely to be interdependent, both directly, as (un)employment and marriage affects the decision to stay, and indirectly, as many observed and unobserved (e.g. risk attitude) student characteristics influence their labour market dynamics, their marriage formation decision and their return migration decision. Assessing the impact of (un)employment spells and marriage on the intensity to leave the country without taking this interdependence into account would bias the results.

We have addressed these issues using a unique Dutch administrative panel of the entire population of the recent (1999–2007) inflow of foreign students into the Netherlands, for which we observe entry, exit, marriage and labour market histories. The large size of the data permitted us to stratify the analysis by five distinct student groups, based on their country of birth. The correlated hazards method enabled us to estimate the effects of (un)employment and marriage histories on migration durations, while we controlled for (correlated) unobserved heterogeneity.

In line with previous research (Kırdar 2009; Bijwaard et al. 2014), the estimation results indicate that when students become unemployed they are more inclined to return. The effect of finding a job on return is more ambiguous. For students from developed (including EU) countries finding a job hardly affects their return. While students from less developed countries and Antilles/Surinam are more prone to leave after finding a job. This seems contrary to intuition, but can be explained by realising that the labour market experience in the Netherlands will give them better job opportunities in their home or a third country. Another explanation for this counterintuitive result is that the students are target savers, assuming that they leave when their accumulated savings exceed some threshold. In our context of foreign students the possibility to save is only conceivable after the student starts working.

Confirming intuition, students who find a partner in the Netherlands are much less inclined to leave. However, this marriage effect is not significant for students from new EU and DC countries. An explanation for this is that these students marry other foreigners and choose to move to either country of origin. Unfortunately, we do not observe any spousal information and are therefore unable to test this. Another issue with the estimated marriage effect is that we only observe official marriages and no cohabitation. We expect that cohabitation is more common among students from other EU countries.

Understanding the link between the labour market behaviour, family formation and return migration decisions also assists policy makers. Return behaviour of students is closely related to the immigration and integration policy of the host. Immigration of students often turns into skilled labour migration, when the student remains in the country working in a highly skilled job. From our analysis we derive that it is not only the question of how to assure that the students come to the Netherlands but also how to retain them. When the Dutch government facilitates the prolonged stay of foreign students, this would increase the number of high-skilled labour migrants in the country, especially in the long-run. The recent (beyond the observation period) introduction of a more extensive job search period in which foreign students are allowed to stay in the country a few months after graduation will probably reduce the effect of becoming unemployed on return of these students. Other possible policies to retain foreign students are providing them better access to affordable real estate, ease labour market access for sectors in demand such as ITC and technology industries and, for those students with a non-Dutch spouse, ease immigration and labour market entry of their spouse.

## Footnotes

1 The Skilled Workers Regulation of 2004 made it possible for PhD students (and postdocs) to be regarded as skilled workers without having to meet the minimum income threshold of €37,574 per annum.

2 The results of sensitivity checks on the length of the spurious employment spells are available upon request.

3 The estimated hazard is calculated a (Epanechnikov) kernel smooth of the difference in the Nelson–Aalen hazard contributions. A statistical test of the equality of the survival functions was rejected.

4 Unemployed in the broad sense of without a (paid) job and not receiving any (official) student grant. Most unemployed students are not eligible for unemployment benefits, and we therefore observe zero income in the Netherlands.

5 We ignore possible divorce as only a few students first marry and then divorce.

6 To assure that the probability is between zero and one we estimate $q_{\mathrm{r}}$ with $p_{\mathrm{r}} = \hbox{e}^{q_{\mathrm{r}}}/(1+\sum \nolimits \hbox{e}^{q_{\mathrm{j}}})$.

7 Because our focus is on the return migration of students, we do not discuss the impact of control variables on the other hazards. These results are available upon request.

8 Available from the authors upon request.
